# Vitamin D Status, Filaggrin Genotype, and Cardiovascular Risk Factors: A Mendelian Randomization Approach

**DOI:** 10.1371/journal.pone.0057647

**Published:** 2013-02-27

**Authors:** Tea Skaaby, Lise Lotte Nystrup Husemoen, Torben Martinussen, Jacob P. Thyssen, Michael Melgaard, Betina Heinsbæk Thuesen, Charlotta Pisinger, Torben Jørgensen, Jeanne D. Johansen, Torkil Menné, Berit Carlsen, Pal B. Szecsi, Steen Stender, Runa Vavia Fenger, Mogens Fenger, Allan Linneberg

**Affiliations:** 1 Research Centre for Prevention and Health, Glostrup Hospital, Glostrup, Denmark; 2 Department of Biostatistics, University of Copenhagen, Copenhagen, Denmark; 3 National Allergy Research Centre, Department of Dermato-Allergology, Copenhagen University Hospital Gentofte, Hellerup, Denmark; 4 Department of Clinical Biochemistry, Copenhagen University Hospital Gentofte, Hellerup, Denmark; 5 Faculty of Health Science, University of Copenhagen, Copenhagen, Denmark; 6 Faculty of Medicine, Alborg University, Alborg, Denmark; 7 Department of Clinical Biochemistry, Hvidovre Hospital, Hvidovre, Denmark; Innsbruck Medical University, Austria

## Abstract

**Background:**

Vitamin D deficiency is associated with increased cardiovascular disease risk in observational studies. Whether these associations are causal is not clear. Loss-of-function mutations in the filaggrin gene result in up to 10% higher serum vitamin D concentrations, supposedly due to a decreased UV-protection of the keratinocytes. We used a Mendelian randomization approach to estimate the causal effect of vitamin D status on serum lipids, blood pressure, body mass index, waist circumference, and the metabolic syndrome.

**Methods:**

Three population based studies were included, Monica10 (2,656 individuals aged 40–71 years), Inter99 (6,784 individuals aged 30–60 years), and Health2006 (3,471 individuals aged 18–69 years) conducted in 1993–94, 1999–2001, and 2006–2008, respectively. Participants were genotyped for the two most common filaggrin gene mutations in European descendants R501X and 2282del4, in all three studies and further for the R2447X mutation in the Inter99 and Health2006 studies. Filaggrin genotype was used as instrumental variable for vitamin D status. Baseline measurements of serum 25-hydroxyvitamin D were performed in all three studies.

**Results:**

Instrumental variable analyses showed a 23.8% (95% confidence interval, CI 3.0, 48.6) higher HDL cholesterol level and a 30.5% (95% CI: 0.8, 51.3) lower serum level of triglycerides per doubling of vitamin D. These associations were, however, not statistically significant when applying the Bonferroni adjusted significance level. The remaining lipids showed non-significant changes in a favorable direction. Doubling of vitamin D gave a non-significantly lower odds ratio = 0.26 (95% CI: 0.06, 1.17) of the metabolic syndrome. There were no statistically significant causal effects of vitamin D status on blood pressure, body mass index, or waist circumference.

**Conclusion:**

Our results support a causal effect of higher vitamin D status on a more favorable lipid profile, although more studies in other populations are needed to confirm our results.

## Introduction

Vitamin D is ingested from the diet and dietary supplements, but the main source of vitamin D is derived from solar radiation of the skin. Vitamin D deficiency is common and has been associated with a number of common diseases such as cardiovascular disease, diabetes, cancer [1] and mortality [2]; [3] in addition to the traditional role in bone metabolism and remodeling. However, vitamin D status is also associated with several behavioral and environmental factors, such as season and smoking habits, potentially biasing the estimates of traditional observational studies; whether the association between vitamin D status and e.g. cardiovascular disease is causal is still not clear.

Mendelian randomization refers to the random allocation of alleles during meiosis [4]. The allocation is expected to be independent of behavioral and environmental factors allowing estimation of unconfounded risk associations that are not due to reverse causation [4]. Mendelian randomization uses genetic variants as instrumental variables (IVs) to estimate the causal effect of phenotypes, such as vitamin D status, on disease-related outcomes, and is believed to overcome unmeasured confounding [5]; [6].

Important determinants of vitamin D status include sun exposure and diet [1], but recently loss-of-function mutations in the filaggrin gene have been shown to result in up to 10% higher serum vitamin D concentrations [7], supposedly due to a decreased UV-protection of the keratinocytes [8]. Mutations affect 8–10% of Northern Europeans [9]; [10], of which 2282del4 and R501X cover about 80% of the known mutations [11]. Filaggrin deficiency causes ichtyosis vulgaris characterized by xerosis, keratosis pilaris and palmar hyperlinearity but also increases the risk of atopic dermatitis, allergic rhinitis, asthma, and food allergies [11]; [12].

We used the filaggrin genotype as an instrumental variable to estimate the causal effect of vitamin D [13] –as assessed by serum 25-hydroxyvitamin D (25-OH-D) concentration–on serum lipids, blood pressure, body mass index (BMI), waist circumference, and the metabolic syndrome in three general population samples.

## Methods

### Ethics Statement

Participants gave their informed written consent, and the studies were approved by the Ethics Committee of Copenhagen and the Danish Data Protection Agency. The recommendations of the Declaration of Helsinki were followed.

### Study Populations

We used the three population based studies, Monica10, Inter99, and Health2006, recruited from the Danish Central Personal Register as random samples of the population in the southern part of the former Copenhagen County. The studies included questionnaires, physical examinations, and blood tests. The Monica10 study is a 10-year follow-up study of the Monica1 study conducted in 1982–1984 and including examinations of 3,785 individuals of Danish origin. The follow-up study (1993–94) included 2,656 individuals between 40–71 years and had a participation rate of 70.2% [14].

The Inter99 study conducted in 1999–2001 included 6,784 individuals aged 30–60 years from a general population [15]. The baseline participation rate was 52.5%. The Inter99 study was a population-based randomized controlled trial (CT00289237, ClinicalTrials.gov) investigating the effects of lifestyle intervention on CVD. Details on the study and the intervention program have been described elsewhere [15]. Only participants with a Northern European origin were included in the current study. Both current and potential former nationalities of participants and their parents were considered (information from registries and self-reported questionnaires). A Northern European origin was defined as a Danish, Norwegian, Swedish, Icelandic, or Faroese nationality.

In the Health2006 study, a sample of 7,931 Danish citizens aged 18 to 69 years, born in Denmark, was invited to a general health examination [12]. A total of 3,471 (43.8%) individuals were examined between June 2006 and June 2008. In total, we included 2,571, 6,096, and 3,316 participants from the Monica10, the Inter99, and the Health2006 study, respectively, with measurements of vitamin D status and filaggrin genotype.

### Vitamin D Measurements

Serum samples from the participants in the Monica10 and the Inter99 studies had been stored at −20°C while the samples from the Health2006 study were stored at −80°C until the analyses of 25-OH-D in 2009, 2010, and 2011, respectively. Measurements of serum 25-OH-D in the Monica10 population were performed using the IDS-SYS 25-Hydroxy Vitamin D method with the IDS-iSYS Multi-Discipline System (IDS Nordic A/S, Herlev, Denmark) [16]. In the Inter99, measurements of 25-OH-D were performed by high performance liquid chromatography (HPLC) [17] as previously described. In the Health2006 study, 25-OH-D was measured by immunoassay using Cobas e411 (Roche Diagnostics, Mannheim, Germany) [7].

### Filaggrin Genotyping

In all three studies, individual regions covering the two most common null mutations of the filaggrin gene, R501X and 2282del4 (in the Inter99 and Health2006 studies also a region covering the R2447X mutation) were amplified from genomic DNA by allele-specific and asymmetric PCR using DNA tagged primers. The obtained PCR products were hybridized to MagPlex C microbeads (Luminex, Austin, Texas) carrying the same tags as DNA probes [18]. Microbeads were subsequently analyzed on a Bio-Plex 200 (Bio-Rad Laboratories, Hercules, Calif). For all three studies, samples also available as DNA were genotyped for filaggrin mutations. The fractions of samples present as DNA were: Monica10∶99.96%; Health06∶99.40%; Inter99 99.95%.

### Outcome

Height and weight were measured without shoes and with light clothes. Body mass index (BMI) was calculated as weight (kg) divided by height (m) squared. Waist circumference was measured between the lowest rib and the iliac crest. Blood pressure (mmHg) was calculated as the average of two measurements in the sitting position.

From fasting blood samples, lipid profile was measured using enzymatic colorimetric methods (Roche, Mannheim, Germany) [9]; [15]; [19]; [20]. Fasting plasma glucose was assessed by the hexokinase/glucose-6-phosphate dehydrogenase assay (Roche Diagnostics, former Boehringer Mannheim, Germany) [15]; [20]; [21].

The metabolic syndrome was defined according to the International Diabetes Foundation [22] as central obesity (waist circumference ≥94 cm for European men and ≥80 cm for European women) and at least two of the following criteria: raised triglycerides (>1.7 mmol/l or specific treatment for this lipid abnormality); reduced HDL-cholesterol (<1.03 mmol/l for men, <1.29 mmol/l for women, or specific treatment for this lipid abnormality); raised blood pressure (systolic ≥130 mmHg, diastolic ≥85 mmHg, or treatment of previously diagnosed hypertension); elevated fasting plasma glucose (≥5.6 mmol/L or previously diagnosed type 2 diabetes).

### Statistical Analyses

Analyses were performed with SAS, version 9.2 (SAS Institute Inc., Cary, NC, USA). Stata/IC 12.1 for Mac (StataCorp, College Station, Texas) was used for the IV analyses with dichotomous outcomes. All p-values were two-tailed, and statistical significance was defined as p<0.05. The Bonferroni method was used to adjust for multiple comparisons. The Bonferroni adjusted significance level was 0.005 (10 outcomes). Individuals were dichotomized into non-mutation carriers or carriers of at least one of the R501X, 2282del4, or R2447X filaggrin mutations. Descriptive characteristics of the participants by filaggrin genotype presented as percent (total number) were compared with the chi-squared test (table 1). We used the SAS procedures proc freq (table 1), proc glm (table 2), proc means and proc glm/logistic (table 3), proc glm/proc logistic for the ordinary regressions (table 4), and proc syslin for the instrumental variable regression of continuous outcomes (table 4). The procedures regress (first stage) and logit (second stage) in Stata were used for the IV analysis with the dichotomous variable metabolic syndrome as outcome (table 4) as outlined in Palmer et al. [23]. To take into account the uncertainty from the first stage regression the sandwich estimator for calculating standard errors was applied [23]. The associations between filaggrin genotype and cardiovascular risk factors are presented in table 3. The Bonferroni adjusted significance level was applied for the regression analyses in tables 3 and 4. The results of the adjusted p-values are reported in the text of the results section.

**Table 1:** Characteristics of study participants according to filaggrin genotype.

Characteristics	% (n)	% (n)	P-value
	Filaggrin genotype	Filaggrin genotype	Chi-square test
	Wildtype	Carrier	
Study			
Monica10	92.4 (2377)	7.6 (194)	0.09
Inter99	91.1 (5555)	8.9 (541)	
Health2006	91.0 (3019)	9.0 (297)	
Gender			
Male	91.2 (5239)	8.8 (503)	0.58
Female	91.6 (5712)	8.5 (529)	
Age, years			
≤45	91.6 (4355)	8.4 (397)	0.58
45–55	91.0 (3588)	9.0 (354)	
≥55	91.5 (3008)	8.5 (281)	
Season of blood collection			
Mar-May	92.0 (2767)	8.0 (239)	0.51
Jun-Aug	91.1 (2447)	8.9 (239)	
Sep-Nov	91.1 (3401)	8.9 (331)	
Dec-Feb	91.3 (2336)	8.7 (223)	
Education			
No	90.7 (1812)	9.3 (187)	0.20
Yes	91.5 (8918)	8.5 (825)	
Body mass index, kg/m^2^			
<18.5	91.5 (140)	8.5 (13)	0.96
18.5–24.9	91.3 (4841)	8.7 (461)	
25–29.9	91.6 (4165)	8.4 (384)	
≥30	91.2 (1800)	8.8 (173)	
Physical activity			
Sedentary	91.5 (2167)	8.5 (201)	0.51
Light	91.2 (6551)	8.8 (631)	
Moderate/vigorous	92.0 (2068)	8.0 (180)	
Weekly intake of fish			
<twice	91.2 (5608)	8.8 (542)	0.46
≥twice	91.6 (5117)	8.4 (471)	
Smoking habits, g/day			
Never smoker	91.1 (3813)	8.9 (374)	0.43
Former smoker	91.2 (3038)	8.8 (292)	
Current smoker, <15	92.4 (1617)	7.6 (132)	
Current smoker, <25	91.6 (1805)	8.4 (166)	
Current smoker, ≥25	90.5 (603)	9.5 (63)	
Alcohol, drinks/week			
0	91.0 (994)	9.0 (98)	0.26
≤7	91.5 (4718)	8.5 (438)	
≤14	92.1 (2339)	7.9 (201)	
>14	90.6 (2417)	9.4 (251)	
Blood pressure lowering medication			
No	91.4 (9913)	8.6 (932)	0.82
Yes	91.2 (1038)	8.8 (100)	
Lipid lowering medication			
No	91.4 (10588)	8.6 (999)	0.84
Yes	91.7 (363)	8.3 (33)	

**Table 2:** Instrumental variable first stage regression i.e. linear regression analyses of the association between the instrumental variable and vitamin D status.

	Number of persons included	Relative difference in % (95% CI)	*P* value	First stage R^2^	First stage F statistic
Filaggrin genotype (Carrier vs. wildtype)					
Combined studies*	11983	7.9 (4.4, 11.6)	<0.0001	0.08	15.1
Monica10	2571	8.8 (2.1, 15.8)	0.009	0.003	6.8
Inter99	6096	6.6 (1.6, 11.9)	0.010	0.001	6.7
Health2006	3316	9.8 (2.9, 17.1)	0.005	0.002	8.0

* Adjusted for study population.

Abbreviations: CI, confidence interval.

**Table 3:** Associations between filaggrin genotype and cardiovascular risk factors.

	Median (IQR) or *% (n)*	Median (IQR) or *% (n)*	Number in regression analyses	Relative difference % or *OR* (95% CI), p-value#
Risk factors	Wildtype	Carrier		Carrier vs. wildtype*
25-OH-D, nmol/l	48.3 (33.2–66.0)	50.7 (36.5–69.9)	10940	7.5 (4.0, 11.1), p<0.0001
Lipids, mmol/l&				
HDL	1.41 (1.16–1.70)	1.45 (1.20–1.72)	10601	2.1 (0.6, 3.7), P = 0.008
LDL	3.50 (2.90–4.20)	3.41 (2.80–4.12)	10492	−1.8 (−3.6, −0.1), P = 0.043
Triglycerides	1.10 (0.80–1.59)	1.06 (0.80–1.50)	10601	−3.5 (−6.5, −0.5), P = 0.024
VLDL	0.50 (0.39–0.70)	0.50 (0.39–0.68)	10496	−3.1 (−5.9, −0.2), P = 0.039
Total cholesterol	5.59 (4.90–6.30)	5.50 (4.80–6.20)	10601	−0.9 (−2.1, 0.3), P = 0.125
Blood pressure, mmHg£				
Systolic	126.5 (116.5–139.0)	127.5 (118.0–138.0)	9940	−0.3 (−1.1, 0.5), P = 0.46
Diastolic	80.0 (74.0–89.0)	80.0 (75.0–89.0)	9940	−0.4 (−1.2, 0.4), P = 0.33
Anthropometrics				
Body mass index, kg/m^2^	25.42 (22.99–28.43)	25.37 (22.89–28.44)	10940	−0.3 (−1.3, 0.8), P = 0.62
Waist circumference, cm	87 (77–96)	87 (77–95)	10931	−0.2 (−1.0, 0.6), P = 0.64
Metabolic syndrome			10931	*0.86 (0.74, 1.0),* P = 0.081
No	*91.24 (7931)*	*8.76 (761)*		
Yes	*91.76 (3005)*	*8.24 (270)*		

# Linear and *logistic* regression, respectively.

& Participants with self-reported use of lipid lowering medication were excluded (n = 396).

£ Participants with self-reported use of blood pressure lowering medication were excluded (n = 1,138).

* Adjusted for study population, age, gender, education, season, intake of fish, physical activity, smoking habits, alcohol consumption, and body mass index (anthropometrics and metabolic syndrome were not adjusted for body mass index).

Abbreviations: CI, confidence interval; IQR, inter quartile range; 25-OH-D, 25-hydroxyvitamin D; OR, odds ratio.

**Table 4:** Ordinary linear/logistic regression and instrumental variable regression of the association between serum 25-OH-D and cardiovascular risk factors.

		Relative difference in % (95% CI)/*OR (95% CI)* per 25-OH-D doubling	Relative difference in % (95% CI)/*OR (95% CI)* per 25-OH-D doubling
Cardiovascular risk factors	Number of individuals included	Ordinary linear/*logistic* regression	Instrumental variable regression¤
Lipids&			
HDL-cholesterol	10601	1.8 (1.2, 2.4), p<0.0001	23.8 (3.0, 48.6), p = 0.02
LDL-cholesterol	10492	−0.3 (−1.0, 0.4), p = 0.36	−16.9 (−31.9, 1.5), p = 0.07
Triglycerides	10601	−2.6 (−3.8, −1.4), p<0.0001	−30.5 (−51.3, −0.8), p = 0.04
VLDL-cholesterol	10496	−1.3 (−2.5, −0.2), p = 0.03	−27.0 (−47.6, 1.8), p = 0.06
Total cholesterol	10601	−0.2 (−0.7, 0.3), p = 0.46	−9.1 (−20.2, 3.5), p = 0.15
Blood pressure£			
Systolic	9940	0.1 (−0.2, 0.4), p = 0.50	−2.8 (−9.9, 4.9), p = 0.47
Diastolic	9940	−0.1 (−0.4, 0.2), p = 0.51	−3.8 (−11.1, 4.1), p = 0.34
Anthropometrics$			
Body mass index	10940	−1.9 (−2.4, −1.6), p<0.0001	−2.5 (−11.8, 7.7), p = 0.62
Waist circumference	10931	−1.8 (−2.1, −1.5), p<0.0001	−1.9 (−9.2, 6.1), p = 0.64
Metabolic syndrome$	10931	*0.78 (0.73, 0.83)*, p<0.0001	*0.26 (0.06, 1.17),* p = 0.08

¤ 2SLS analyses of the association between vitamin D status and cardiovascular risk factors using filaggrin genotype as instrument for vitamin D status.

& Both OLS and IV models were adjusted for study population, age, gender, education, season, intake of fish, physical activity, smoking habits, alcohol consumption, and body mass index. Participants with self-reported use of lipid lowering medication were excluded (n = 396).

£ Both OLS and IV models were adjusted for study population, age, gender, education, season, intake of fish, physical activity, smoking habits, alcohol consumption, and body mass index. Participants with self-reported use of blood pressure lowering medication were excluded (n = 1,138).

$ Both OLS and IV models were adjusted for study population, age, gender, education, season, intake of fish, physical activity, smoking habits, and alcohol consumption.

Abbreviations: CI, confidence interval; 25-OH-D, 25-hydroxyvitamin D; OR, odds ratio.

Serum 25-OH-D concentrations were log2-transformed (used as outcome in the first stage regression) and the continuous outcomes were log-transformed to meet requirements on normality. For the outcomes of interest, we did both ordinary least squares (OLS) regression and two stage least squares (2SLS) regression. The OLS and 2SLS regressions were adjusted for gender; age (≤45, 45–55, or ≥55 years); study cohort (Monica10, Inter99, or Health2006); season of blood sample (March-May, June-August, September-November, or December-February); education/vocational training assessed by the question: “Do you have vocational training?” (no education, education including students); intake of fish (< twice a week, ≥ twice a week); physical activity during leisure time (sedentary, light, or moderate/vigorous); smoking habits (never smokers, ex-smokers, current and occasional smokers <15 g/day; 15–<25 g/day, or ≥25 g of tobacco/day; 1 cigarette = 1 g, 1 cheroot = 2 g, 1 cigar = 3 g, pipe = stated in g); and alcohol consumption (0, >0–7, >7–14, or >14 drinks per week). The analyses of lipids and blood pressure were also adjusted for BMI (<18.5 kg/m^2^, ≥18.5–25 kg/m^2^, ≥25–30 kg/m^2^, and ≥30 kg/m^2^). Further, we excluded participants with self-reported use of lipid lowering medication (n = 396) and participants with self-reported use of blood pressure lowering medication (n = 1,138) in the analyses of lipids and blood pressure as outcomes, respectively.

In the first stage of the IV analyses, the log2-transformed vitamin D status is regressed on our instrument, the dichotomized filaggrin genotype, and the observed covariates. In the second stage, the outcome of interest is regressed (log(HDL), log(LDL), log(VLDL), log(triglycerides), log(total cholesterol), log(BMI), log(waist circumference), log(systolic blood pressure), log(diastolic blood pressure), or metabolic syndrome) on the predicted values from the first-stage regression and the same observed covariates as for the first stage regression. The regression coefficient of the predicted values from the second stage can be interpreted as the causal effect per doubling of vitamin D on the outcome. For continuous outcomes, the regression coefficients were back-transformed and reported as percent with 95% confidence intervals (CI). For dichotomous outcomes, the estimate is only an approximation of the causal effect [24].

## Results

The percentages (number) of carriers of the R501X, 2282del4, and the R2447X filaggrin mutations were 3.3% (394), 4.6% (557), and 1.0% (92) respectively, and the percentage (number) of carriers of at least one of the filaggrin mutations was 8.6% (1,032). The distribution of the genotypes was not associated with the covariates –except for vitamin D– including gender, age, and study cohort (table 1). Further, neither of the genotypes deviated significantly from the expected frequencies under assumption of Hardy-Weinberg equilibrium. The strength of the instrument is assessed in table 2 [25]. The median vitamin D levels (inter quartile range) were 60.9 (44.7–80.8), 48.0 (33.0–65.0), and 41.2 (29.2–55.2) nmol/l in the Monica10, the Inter99, and the Health2006 study, respectively. Being a filaggrin mutation carrier was significantly associated with a higher vitamin D status in all three cohorts. The lipids, blood pressures, BMI, and waist circumference levels were comparable across study populations (data not shown).

In multivariable regression analyses, filaggrin mutation carrier status was significantly associated with a higher HDL level, and lower LDL, triglyceride, and VLDL levels (table 3). These associations were not statistically significant when applying the Bonferroni adjusted significance level. While the negative association between filaggrin mutation carrier status and metabolic syndrome reached borderline significance before Bonferroni adjustments, there were no statistically significant associations between filaggrin mutation carrier status and blood pressure (systolic or diastolic), BMI or waist circumference.

The ordinary regression analyses, OLSs, showed significant associations between vitamin D status and HDL, triglycerides, VLDL, BMI, waist circumference, and metabolic syndrome (table 4) in the same favorable direction as the 2SLS estimates (table 4). When applying the Bonferroni adjusted significance level, only the associations between vitamin D and HDL-cholesterol, triglycerides, BMI, waist circumference, and metabolic syndrome remained statistically significant.

In general, the unadjusted (data not shown) and adjusted two stage least squares, 2SLS, estimates were very similar. The multivariable 2SLS regression analyses showed a 23.8% (95% confidence interval, CI: 3.0, 48.6) higher HDL cholesterol level and a 30.5% (95% CI: 0.8, 51.3) lower serum level of triglycerides per doubling of vitamin D. These associations were, however, not statistically significant when applying the Bonferroni adjusted significance level. The remaining lipids showed non-significant changes in a favorable direction. A doubling of vitamin D gave a non-significantly lower odds ratio (OR) of 0.26 (95% CI: 0.06, 1.17) of the metabolic syndrome. Compared to the OLS estimates, the 2SLS estimates of the effect of vitamin D on lipids were five to tenfold higher. Importantly, the effects of the filaggrin genotype on lipids were very consistent for the cohorts when analyzed separately (data not shown).

There were no statistically significant causal effects of vitamin D status on blood pressure, body mass index, or waist circumference. The OLS and 2SLS estimates of the anthropometric measurements were very similar but only the OLS associations were statistically significant. Excluding the R2447X mutation from the analyses did not change the results substantially.

## Discussion

We found a causal association between vitamin D status and HDL cholesterol and triglycerides when using the filaggrin genotype as an IV for vitamin D status, although not statistically significant when applying the Bonferroni adjusted significance level. Vitamin D status seemed to be associated with a more favorable lipid profile overall, strengthening causal inference between vitamin D status and lipid profile. Further, we replicated the association between filaggrin genotype and vitamin D status reported by Thyssen et al [7] in the Inter99 population. The inverse association between vitamin D status and metabolic syndrome was statistically non-significant. On the other hand, we found no association between vitamin D status and blood pressure, BMI, or waist circumference. However, the confidence intervals are relatively wide and do not exclude a causal effect of vitamin D indicating that large studies are needed to exclude causal effects of vitamin D on these traits.

A recent mendelian randomization study found no significant associations between vitamin D associated SNPs and systolic blood pressure, BMI, total cholesterol, or triglycerides after adjustments [26]. Our results extend these findings regarding diastolic blood pressure, waist circumference, metabolic syndrome, HDL-, LDL- and VLDL-cholesterol. Our results regarding serum lipids are in line with both the results from cross-sectional studies which have found a higher vitamin D level to be associated with a favorable lipid profile [27]–[31] and prospective studies which have shown an inverse association between vitamin D status and triglycerides [28]; [32]. However, the evidence from the few randomized controlled trials (RCTs) examining a possible effect of vitamin D supplementation on lipid profile is inconclusive [29]; [33]–[36]. Jorde et al summarized the results from 10 placebo-controlled double-blind intervention studies with vitamin D supplementation as divergent. They found some studies showing a positive and some a negative effect of vitamin D supplementation. None of the intervention studies were, however, designed for evaluating the relation between vitamin D and lipids, and they were all underpowered [29].

Regarding the observed lack of association between vitamin D status and blood pressure, a meta-analysis on RCTs of vitamin D supplements and blood pressure found weak evidence to support a small effect of vitamin D supplementation on lowering the blood pressure in hypertensive patients [37] whereas another meta-analysis found a non-significant reduction of the systolic blood pressure [38]. As for the observed lack of association with BMI and waist circumference, it may be speculated whether the inverse association with vitamin D status seen in traditional observational studies [39]; [40] can be explained by the fact that the fat soluble vitamin D is sequestered in the adipose tissue resulting in lower levels in obese individuals [41], i.e. that obesity causes low vitamin D status. Given a larger or older study sample, we might have been able to find an association for both blood pressure and anthropometrics.

While the mechanism by which vitamin D could affect the lipid profile is unclear, it may be due to suppression of parathyroid hormone (PTH) secretion by vitamin D, since PTH can reduce lipolysis [42]. Alternatively, vitamin D may increase calcium level, thereby reducing hepatic triglyceride formation and secretion [43]. Finally, vitamin D may have an effect on insulin secretion and sensitivity [44].

The estimates from the 2SLS are higher than the estimates from the OLS. As Mendelian randomization estimates are based on life course differences in the exposure –here vitamin D status–effect estimates can be larger than those derived from traditional observational estimates. Also, the approach avoids the underestimation of risk associations caused by regression dilution in traditional prospective studies [45]. However, sometimes the most recent exposure is the strongest determinant as for the impact on cholesterol levels where a quick response to a raise or decline in a determinant can be expected.

The strengths of our study are the large samples of the general population; the ethnic homogeneity which enables genetic association studies; the detailed information on covariates; the objective measurements of instrument, exposure, and outcome; and the Mendelian randomization approach which has the potential to avoid some of the limitations of observational epidemiology (confounding, reverse causality, and regression dilution bias) for making causal inferences.

Unmeasured confounders could be factors such as sun exposure or dietary habits. Compared to RCTs, Mendelian randomization studies can be done in a representative sample with no required random treatment allocation. Certain limitations of RCTs, such as limited generalizability, high costs, feasibility and ethics, also make the Mendelian randomization approach attractive [46].

Using a genetic variant as proxy for vitamin D status is supposed to give better causal inferences for several reasons. First, unlike vitamin D status, genetic variants are generally not associated with the behavioral, social, and physiological factors that confound the association between vitamin D and cardiovascular risk factors. Second, genetic variants associated with vitamin D status will not be influenced by the onset of disease, and the estimates will therefore be less biased by reverse causation. Third, often a genetic variant will indicate long-term levels of exposure and will not suffer from the measurement error inherent in phenotypes that have high levels of variability like vitamin D status [47]. The estimates from the analyses can be interpreted as the causal effect of vitamin D status on the outcome if the instrument is correlated with vitamin D status; is independent of the unmeasured confounders; and only affects the outcome through vitamin D given the unmeasured confounders [6]. Regarding the dichotomous outcome metabolic syndrome, the estimate from the two-stage procedure is only an approximation of the causal OR. Palmer et al has suggested several possibilities of estimating the causal OR [23]. We also did the suggested probit analysis and got comparable results (p = 0.03 as compared to the reported p = 0.08, table 4).

The limitations of the study are the risk of selection bias if filaggrin genotypes were unequally distributed among the persons who died before the study; and the heterogeneity of vitamin D measurements. Importantly, the effects of the filaggrin genotype on serum lipids in particular were very consistent for the cohorts when analyzed separately. Although Mendelian randomization is a potentially powerful technique for strengthening causal inference, several issues could violate the IV assumptions: canalization i.e. developmental changes compensating for genetic variation; linkage disequilibrium between filaggrin genotype and other causal variants; pleiotropy which refers to a single gene having multiple biological functions [25]; and epigenetic effects i.e. non-Mendelian, heritable changes in gene expression not accompanied by changes in DNA sequence [5]; [46]. Given an inheritance of gene expression from one’s parents, we also need to assume a random distribution of epigenetic changes at conception to comply with the core assumptions of the Mendelian randomization methodology. The analyses are based on the assumption that filaggrin genotype only affects cardiovascular risk factors through vitamin D status.

The implication of using three mutations in the Inter99 and Health2006 studies while only using 2 in the Monica10 study needs consideration. The R2447X mutation is rare compared to the two other mutations, and the effects of all three FLG mutations are supposed to be the same [8]. Further, excluding the R2447X mutation from the analyses did not change the results.

25-OH-D concentrations are different between the studies. This could be due to several factors such as the different methods for measuring vitamin D, evaporation during storage, or a real decrease in vitamin D levels in the population over the years. It is recommended to store samples for measurement of 25-OH-D at -80°C but studies have demonstrated stability of 25-OH-D in serum samples under different conditions [48]; [49]. In general, measuring serum 25-OH-D is associated with methodological concerns, and variations between methods are considerable [50]. Differences between methods are well known and variations among laboratories using the same method or assay are significant. We adjusted for the method of measuring/different levels of vitamin D by adjusting for study population. Results of analyses for each cohort separately were consistent with the combined analyses of the three cohorts which indicate that methodological differences between the cohorts did not influence our results substantially.

We investigate several outcomes/hypotheses, and it should be considered whether multiple testing represents a concern. Although we had some a priori evidence to support our hypotheses, we provided the Bonferroni adjusted significance level along with the traditional level of significance to decrease the risk of false-positive results (type 1 error). When p-values were adjusted for multiple testing by the Bonferroni method, none of the IV associations remained statistically significant emphasizing that our results need confirmation in other populations.

In conclusion, our results support a causal effect of higher vitamin D status on a more favorable lipid profile and possibly a beneficial effect in the development of the metabolic syndrome, although our results need to be confirmed in other studies. Further, we replicated the results from a previous study reporting a higher vitamin D status among filaggrin mutation carriers. A key issue in instrumental variable analyses is having a sufficiently strong instrument. Filaggrin genotype and other vitamin D related genetic variants only explain a small proportion of the variance in the observed vitamin D levels compared to the variance explained by strong determinants such as sun exposure and diet, and future research should focus on developing more efficient IV tools, e.g. by including more genetic determinants of vitamin D status or even a genetic risk score based on several SNPs.
